# Supplementary material for: Somatic Mutations Alter Interleukin Signaling Pathways in Grade II Invasive Breast Cancer Patients: An Egyptian Experience
Source: Curr Issues Mol Biol. 2022 Nov 26;44(12):5890–901. doi: 10.3390/cimb44120401 (PMC9777163; doi:10.3390/cimb44120401)
Supplement: Supplementary file 1 [file cimb-44-00401-s001.zip › supplementary 1.pdf]

ion torrent

by life technologies™

Ion AmpliSeq™ Comprehensive Cancer Panel target gene list

The Ion AmpliSeq™ Comprehensive Cancer Panel targets the exons of 409 tumor suppressor genes and oncogenes frequently cited and frequently mutated. Strategically designed to interrogate coding DNA sequences and splice variants across multiple gene families simultaneously, our pathway-based gene selection profiles the mutational spectrum in cancer driver genes and drug targets along with signaling cascades, apoptosis genes, DNA repair genes, transcription regulators, inflammatory response genes, and growth factor genes in a single assay. Additionally, this panel targets all 50 genes targeted in the focused Ion AmpliSeq™ Cancer Panel v2; these genes are indicated in dark grey cells.

**ABL1**	AURKA	BMPR1A	CDK4	**CTNNB1**	EPHB4	FANCD2	FZR1
ABL2	AURKB	**BRAF**	CDK6	CYLD	EPHB6	FANCF	G6PD
ACVR2A	AURKC	BRD3	CDK8	CYP2C19	**ERBB2**	FANCG	GATA1
ADAMTS20	AXL	BRIP1	**CDKN2A**	CYP2D6	ERBB3	FAS	GATA2
AFF1	BAI3	BTK	CDKN2B	DAXX	**ERBB4**	**FBXW7**	GATA3
AFF3	BAP1	BUB1B	CDKN2C	DCC	ERCC1	**FGFR1**	GDNF
AKAP9	BCL10	CARD11	CEBPA	DDB2	ERCC2	**FGFR2**	**GNA11**
**AKT1**	BCL11A	CASC5	CHEK1	DDIT3	ERCC3	**FGFR3**	**GNAQ**
AKT2	BCL11B	CBL	CHEK2	DDR2	ERCC4	FGFR4	**GNAS**
AKT3	BCL2	CCND1	CIC	DEK	ERCC5	FH	GPR124
**ALK**	BCL2L1	CCND2	CKS1B	DICER1	ERG	FLCN	GRM8
**APC**	BCL2L2	CCNE1	CMPK1	DNMT3A	ESR1	FLI1	GUCY1A2
AR	BCL3	CD79A	COL1A1	DPYD	ETS1	FLT1	HCAR1
ARID1A	BCL6	CD79B	CRBN	DST	ETV1	**FLT3**	HIF1A
ARID2	BCL9	CDC73	CREB1	**EGFR**	ETV4	FLT4	HLF
ARNT	BCR	**CDH1**	CREBBP	EML4	EXT1	FN1	**HNF1A**
ASXL1	BIRC2	CDH11	CRKL	EP300	EXT2	FOXL2	HOOK3
ATF1	BIRC3	CDH2	CRTC1	EP400	**EZH2**	FOXO1	**HRAS**
**ATM**	BIRC5	CDH20	**CSF1R**	EPHA3	FAM123B	FOXO3	HSP90AA1
ATR	BLM	CDH5	CSMD3	EPHA7	FANCA	FOXP1	HSP90AB1
ATRX	BLNK	CDK12	CTNNA1	EPHB1	FANCC	FOXP4	ICK

Learn more about the Ion AmpliSeq™ Comprehensive Cancer Panel at lifetechnologies.com/ampliseq

life technologies™

Ion AmpliSeq™ Comprehensive Cancer Panel target gene list

**IDH1**	**KRAS**	**MLH1**	NFKB2	PIK3C2B	RARA	SOCS1	TOP1
**IDH2**	LAMP1	MLL	NIN	**PIK3CA**	**RB1**	SOX11	**TP53**
IGF1R	LCK	MLL2	NKX2-1	PIK3CB	RECQL4	SOX2	TPR
IGF2	LIFR	MLL3	NLRP1	PIK3CD	REL	**SRC**	TRIM24
IGF2R	LPHN3	MLLT10	**NOTCH1**	PIK3CG	**RET**	SSX1	TRIM33
IKBKB	POT1	MMP2	NOTCH2	PIK3R1	RHOH	**STK11**	TRIP11
IKBKE	LPP	MN1	NOTCH4	PIK3R2	RNASEL	STK36	TRRAP
IKZF1	LRP1B	**MPL**	**NPM1**	PIM1	RNF2	SUFU	TSC1
IL2	LTF	MRE11A	**NRAS**	PKHD1	RNF213	SYK	TSC2
IL21R	LTK	MSH2	NSD1	PLAG1	ROS1	SYNE1	TSHR
IL6ST	MAF	MSH6	NTRK1	PLCG1	RPS6KA2	TAF1	UBR5
IL7R	MAFB	MTOR	NTRK3	PLEKHG5	RRM1	TAF1L	UGT1A1
ING4	MAGEA1	MTR	NUMA1	PML	RUNX1	TAL1	USP9X
IRF4	MAGI1	MTRR	NUP214	PMS1	RUNX1T1	TBX22	**VHL**
IRS2	MALT1	MUC1	NUP98	PMS2	SAMD9	TCF12	WAS
ITGA10	MAML2	MUTYH	PAK3	POU5F1	SBDS	TCF3	WHSC1
ITGA9	MAP2K1	MYB	PALB2	PPARG	SDHA	TCF7L1	WRN
ITGB2	MAP2K2	MYC	PARP1	PPP2R1A	SDHB	TCF7L2	WT1
ITGB3	MAP2K4	MYCL1	PAX3	PRDM1	SDHC	TCL1A	XPA
JAK1	MAP3K7	MYCN	PAX5	PRKAR1A	SDHD	TET1	XPC
**JAK2**	MAPK1	MYD88	PAX7	PRKDC	SEPT9	TET2	XPO1
**JAK3**	MAPK8	MYH11	PAX8	PSIP1	SETD2	TFE3	XRCC2
JUN	MARK1	MYH9	PBRM1	PTCH1	SF3B1	TGFBR2	ZNF384
KAT6A	MARK4	NBN	PBX1	**PTEN**	SGK1	TGM7	ZNF521
KAT6B	MBD1	NCOA1	PDE4DIP	PTGS2	SH2D1A	THBS1	
KDM5C	MCL1	NCOA2	PDGFB	**PTPN11**	SMAD2	TIMP3	
KDM6A	MDM2	NCOA4	**PDGFRA**	PTPRD	**SMAD4**	TLR4	
**KDR**	MDM4	NF1	PDGFRB	PTPRT	SMARCA4	TLX1	
KEAP1	MEN1	NF2	PER1	RAD50	**SMARCB1**	TNFAIP3	
**KIT**	**MET**	NFE2L2	PGAP3	RAF1	**SMO**	TNFRSF14	
KLF6	MITF	NFKB1	PHOX2B	RALGDS	SMUG1	TNK2	

Learn more about the Ion AmpliSeq™ Comprehensive Cancer Panel at lifetechnologies.com/ampliseq

life technologies™

FOR RESEARCH USE ONLY. NOT INTENDED FOR DIAGNOSTIC PROCEEDURES.

©2012 Life Technologies Corporation. All rights reserved. The trademarks mentioned herein are the property of Life Technologies Corporation and/or its affiliate(s) or their respective owners. **CO25560 0812**
